# *Bacillus velezensis* A2 Inhibited the Cecal Inflammation Induced by Zearalenone by Regulating Intestinal Flora and Short-Chain Fatty Acids

**DOI:** 10.3389/fnut.2022.806115

**Published:** 2022-03-03

**Authors:** Jing Cai, Nan Wang, Jia Chen, Aibo Wu, Eugenie Nepovimova, Martin Valis, Miao Long, Wenda Wu, Kamil Kuca

**Affiliations:** ^1^Key Laboratory of Zoonosis of Liaoning Province, College of Animal Science and Veterinary Medicine, Shenyang Agricultural University, Shenyang, China; ^2^MOE Joint International Research Laboratory of Animal Health and Food Safety, College of Veterinary Medicine, Nanjing Agricultural University, Nanjing, China; ^3^SIBS-UGENT-SJTU Joint Laboratory of Mycotoxin Research, CAS Key Laboratory of Nutrition, Metabolism and Food Safety, Shanghai Institute of Nutrition and Health, Chinese Academy of Sciences, University of Chinese Academy of Sciences, Shanghai, China; ^4^Department of Chemistry, Faculty of Science, University of Hradec Kralove, Hradec Kralove, Czechia; ^5^Department of Neurology, Faculty of Medicine and University Hospital Hradec Kralove, Charles University, Hradec Kralove, Czechia; ^6^Biomedical Research Center, University Hospital Hradec Kralove, Hradec Kralove, Czechia

**Keywords:** zearalenone, *Bacillus velezensis* A2, intestinal flora, inflammatory, short-chain fatty acid

## Abstract

Zearalenone (ZEA) as an estrogen-like mycotoxin can cause the inflammatory injury of the cecum. How to reduce the harm that ZEA causes to humans and animals is a current concern for researchers. In this study, we aimed to ascertain whether *Bacillus velezensis* A2 (A2) could alleviate injury caused by ZEA by regulating the intestinal flora and the content of short chain fatty acids in the cecum among mice. Our results showed that *Bacillus velezensis* A2 improved the fold height, myometrial thickness, and crypt depth of the cecum induced by ZEA. Enzyme-linked immunosorbent assay and Western blotting results showed that A2 could decrease the ZEA-induced increase in expression levels of IL-2, IL-6, IFN-γ, TNF-α, and FC. Studies also showed that A2 increased the content of SCFA in the cecum which was decreased by ZEA. The microbial communities in the cecum were changed when given ZEA or A2. A2 was found to greatly reduce the ZEN-induced increase in the relative abundance of *p_Actinobacteria*, *p_Protebacteria*, *o_Coriobacteriales*, *g_Anaerotruncus*, *g_Pseudoflavonifractor*, *g_Lachnoclostridium*, *g_Enterorhabdus*, and *f_Oscillospiraceae*, and increase the ZEN-induced decrease in the relative abundance of *f_Coriobacteriales*. Results indicated that *Bacillus velezensis* A2 can largely ameliorate the intestinal inflammatory injury induced by ZEA in mice by regulating the microflora and short chain fatty acids content.

## Introduction

Zearalenone is mainly produced by a variety of *Fusarium fungi*, such as *F. culmorum*, *F. graminearum*, *F. crookwellense*, *F. oxysporum*, and *F. equiseti*, and it contaminates corn, oats, rice, wheat, barley, and other cereal crops (1–3). The gastrointestinal tract (GIT) will be the first organ where the toxin interacts with the animal after ingestion of ZEA (4), part of the toxin will be excreted by the kidney, rest will continue to accumulate in the organ, resulting in renal toxicity, then hepatotoxicity upon accumulation in the liver, *etc*. It can also cause low fertility, immune system disorders, and genotoxicity (5). Therefore, developing a safe and effective method to decrease the toxicity of ZEA and promote its metabolism to reduce the cost of feed and livestock production has become a focus of much research.

There are many microorganisms in the human intestine: exploring the interaction between microorganisms and their host has research value in evaluating the relationship between microorganisms and intestinal diseases (5). For example, microbes can prevent pathogens from settling in the gut, protect the intestinal barrier capacity, and regulate the associated lymphoid tissue and immune function in the gut (7). Use of beneficial microbes to protect animals from colonization and infection by intestinal pathogens is known as ‘colonization resistance,’ which arises from the interaction between intestinal microbes and the mucosal surface, epithelial cells, and immune system (8). Therefore, it has gradually become a new trend in research and development to find edible probiotics as food additives to protect the intestinal tract from infection by pathogenic microorganisms and improve human health.

*Bacillus* is a probiotic that can be used to remove mycotoxins and has great potential for use as a feed additive (9). *Bacillus* promotes intestinal health by producing antimicrobial peptides to inhibit the toxicity of bacterial pathogens (10). It can also promote intestinal flora to produce beneficial metabolites to regulate intestinal health, and even play an essential role in improving body’s immune system and stimulating body growth (10).

Homeostasis of gut microbes plays a key regulatory role in prevention of animal diseases and health (6). Gut microbes can also accelerate the normal development of the GIT and promote nutrient digestion and absorption (11). This is because some carbohydrates that cannot be consumed and absorbed in the small intestine are produced by intestinal microorganisms under anaerobic fermentation to produce short chain fatty acids (SCFA), mainly including acetate, propionate, and butyrate, providing 10% of the daily requirement thereof (12). Some of these SCFA productions are beneficial to the host, affecting the immune and metabolic systems of the host, improving intestinal barrier function, reducing intestinal inflammation, and even affecting the brain function of the host (13).

In this study, mouse models were used to judge whether A2 has a protective effect on the inflammatory injury induced by ZEA. In the experiment, by detecting the SCFA content and the intestinal flora in cecal, analyze whether the change of SCFA content in cecal is related to ZEA induced inflammatory injury, and judge whether the intestinal flora is involved in the mitigation process of the ZEA poisoning damage, so as to provide theoretical support for probiotics to regulate the health of intestinal flora.

## Materials and Methods

### Experimental Animals

Forty 4-week-old male Kunming mice (25 ± 2 g) were selected and reared for 28 days. Mice were protected from stress caused by feeding, by ensuring that there were four or five mice per feed box. Before the formal test begins, the mice were allowed a 1-week adaptation period. During the experiment, the SPF laboratory animal care principles were strictly followed, the experiments were permitted by the Shenyang Agricultural University under the Laboratory Animal Care Ethics Committee (People’s Republic of China Animal Ethics Regulations and Guidelines) for animal experiments (Permit No. 264SYXK2011-0001, September 2018). The mice were given a free diet and were kept at 45–55% relative humidity, a temperature of 23 ± 2°C, and provided with equal amounts of light and dark (12 h: 12 h *per diem*) in freely circulating air.

### Experimental Design

Forty mice were randomly divided into four groups: a blank control group, ZEA toxin group, A2 strain group, and detoxification group, which were denoted as the CON, ZEA, A2, and A2 + ZEA groups, respectively. The LB medium used in the experiment was purchased from Solarbio (Beijing, China). The A2 strain was isolated and preserved in the laboratory of Shenyang Agricultural University, China. The A2 strain is a *Bacillus*: the bacteria are short rod-shaped, and irregular in arrangement, and can be visualized as malachite green-dyed spores. In this experiment, 40 mg/kg BW ZEA was used as the experimental dosage, 20.0 mg ZEA (American Sigma) was dissolved in 1.0 mL ethanol and stored in a refrigerator at −20°C in the dark as a ZEA storage solution. The working solutions of the CON group (2 mL sterile LB culture medium) and ZEA group (0.5 mL ZEA reserve solution and 1.5 mL LB culture medium) were configured beforehand. The working fluids of group A2 (2 mL of A2 strain fermentation broth cultured for 24 h) and group A2 + ZEA (0.5 mL of ZEA reserve solution and 1.5 mL of A2 strain fermentation broth cultured for 24 h) were used, and the gavage dose of each mouse was maintained at 0.2 mL. At 28 days, the mice were sacrificed, and the cecal tissues and feces were collected for follow-up tests.

### Histological Assessment of Cecal Mucosa

At the end of the experiment, five mice were randomly selected from each group, the abdominal cavity was cut open, and a 20-mm long sample was quickly taken from the cecal tissue. The cecal tissue specimens were cleaned and fixed in 10% formalin fixative. The pathological changes in the cecal tissues were photographed and observed by dehydration, transparency, paraffin embedding, sectioning (5 μm), H & E staining (Abcam), and Alcian Blue (AB) staining (Abcam). Finally, photographs were taken under the microscope at 200 × magnification, under consistent background lighting to ensure that the specimens filled the field of vision. Image-pro Plus 6.0 software was used to measure the fold height, myometrial thickness, and crypt depth (mm), respectively.

### Detection of Inflammatory Indicators in the Cecum of Mice

Serum and stool samples were collected from mice, with each set of data coming from at least five independent replicates. Inflammatory markers, including IL-2, IL-6, IFN-γ, TNF-α, and fecal calprotectin (FC) in fecal samples, were measured in accordance with the ELISA kit instructions.

### Detection of Inflammatory Factors by Western Blot Assay

Total protein was extracted from mouse cecum using Solarbio (Beijing, China) total protein extraction kit, and measured using a protein quantification kit (TransGen Biotech, Beijing, China); protein concentration and protein loading were determined. Electrophoresis was performed in the electrophoretic solution using PAGE gel and then transferred to the PVDF membrane. The PVDF membrane was sealed in skimmed milk powder at room temperature for 45 min, then transferred to the corresponding primary antibody, sealed, and stored at 4°C overnight. Among them, IL-2, IL-6, IFN-γ, and TNF-α were purchased from ThermoFisher, and the proportion of use was in accordance with the ratios recommended in the instructions supplied therewith. The next day, the PVDF membrane was washed in TBST, and the secondary antibody was enclosed at room temperature for 2 h. Finally, the DNR chemiluminescence gel imaging system was used for color development.

### Detection of Short Chain Fatty Acids in Mouse Feces

Fresh mouse feces (about 0.5 g) were collected and investigated by high-performance liquid chromatography (HPLC). The procedure was as follows: we put fresh feces in a centrifugal tube and added 2 mL 0.5-mmol/L H_2_SO_4_, mixed it evenly through an oscillator, and centrifuged it at 13,400*g*. After centrifugation, 1.5 mL of supernatant was absorbed, and samples were loaded by HPLC. The retention time and peak area were recorded, and the content of SCFA was calculated after drawing the standard curve. The HPLC conditions included detection column (Agilent Eclipse XDB-C18 4.6 × 250 mm, 5 μm); liquid system flow rate: 0.7 mL/min; column temperature: 30°C; injection volume: 20.00 μL; the excitation wavelength and emission wavelength of the fluorescence detector were 274 and 440 nm, respectively. Mobile phase: mobile phase A consisted of 0.1% formic acid aqueous solution, and mobile phase B consisted of chromatographic grade methanol.

### Analysis of Cecum Flora by 16S rDNA Sequencing

To ensure the efficiency and quality of DNA extraction from intestinal contents, the E.Z.N.A.^®^ Stool DNA Kit (D4015, Omega, Inc., Norcross, GA, United States) were used to extract DNA from four sets of samples, each of which includes seven independent repeats. The reagents used to extract DNA from samples were effective in preparing DNA from most bacteria. The V4 region of the prokaryotic (bacterial and archaeal) small-subunit (16S)rRNA gene was amplified with slightly modified versions of primers 515F(5′-GTGYCAGCMGCCGCGGTAA-3′) and 806R(5′-GGACTACNVGGGTWTCTAA-3′) (14). The 5′ ends of the primers were tagged with specific barcodes per sample and sequencing universal primers.

Polymerase Chain Reaction (PCR) amplification was carried out in a total volume of 25 μL reaction mixture containing 25 ng of template DNA, 12.5 μL PCR Premix, 2.5 μL of each primer, and PCR-grade water to adjust the volume. The PCR conditions to amplify the prokaryotic 16S fragments included: an initial denaturation at 98°C for 30 s; 35 cycles of denaturation at 98°C for 10 s, annealing at 54°C/52°C for 30 s, and extension at 72°C for 45 s; then final extension at 72°C for 10 min. The PCR products were validated using 2% agarose gel electrophoresis. The PCR products were purified by AMPure XT beads (Beckman Coulter Genomics, Danvers, MA, United States) and quantified by Qubit (Invitrogen, Carlsbad, CA, United States). The amplicon pools were prepared for sequencing. The size and quantity of the amplicon library were assessed on Agilent 2100 Bioanalyzer (Agilent, Palo Alto, CA, United States) and with the Library Quantification Kit for Illumina (Kapa Biosciences, Woburn, MA, United States), respectively.

According to the manufacturer’s recommendations (provided by LC-Bio), samples were sequenced on an Illumina MiSeq platform. Sequences with a similarity greater than, or equal to 97% were assigned to the same operational taxonomic units (OTUs) by Versearch (v2.3.4). Alpha diversity was applied in analysis of the complexity of species diversity for a sample through four indices: Chao1, Shannon, Simpson, and Observed species. All these indices in our samples were calculated with QIIM (Version 1.8.0). Beta diversity analysis was conducted to ascertain the differences of samples in terms of species complexity. Beta diversity was obtained by principal coordinates analysis (PCoA) and cluster analysis by QIIME software (Version 1.8.0). The raw sequencing data generated from this study have been deposited NCBI SRA^1^ under the BioProject accession number PRJNA773604.

### Data Statistics

All the data in this study were derived from at least three independent replicates, and the data results were expressed in the form of mean ± standard deviation (X ± SD). Statistical analysis software (SPSS 20.0) was employed to conduct correlation analysis of the results, and the differences among groups were compared by one-way analysis of variance, where *P* < 0.05 verified that the data are statistically significant. The histogram statistical graphs in this experiment were drawn by Graphpad Prism 8.0. “*” and “^**^” indicate comparisons with ZEA and CON groups, and “#” and “##” denote a comparison with the ZEA group.

## Test Results

### H & E Staining Results of the Cecum

**FIGURE 1:**
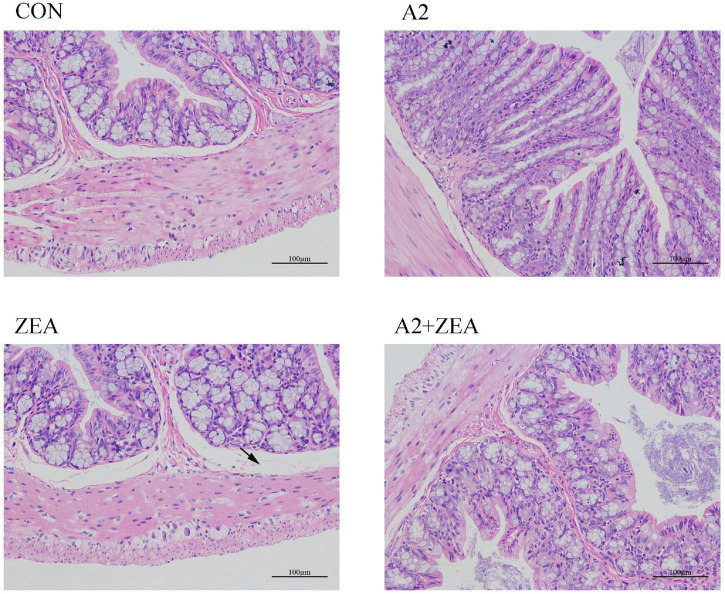
H & E image of cecal tissue (X200).

As shown in Figure 1, in the CON group, the intestinal epithelial structure of the mucosal layer was shown to be intact, the morphology and structure of the epithelial cells were normal and tightly arranged. There were abundant intestinal glands of the lamina propria, with more cup cells visible, and no obvious inflammation was observed. In the ZEA group, the structure of the intestinal glands was disorganized, and the thickness of the intestinal mucosa muscle layer was inconsistent. Compared with the CON group, the ZEA group was obviously thinner, and more cup cells could be visible. Meanwhile, a series of edema in the submucosa and a loose arrangement of connective tissue (black arrow) were found. Compared with the ZEA group, the cecal tissue structure in the A2 + ZEA group was remarkably improved, and the submucosal edema was significantly reduced, which is close to the normal group. These results imply that A2 can ameliorate intestinal damage caused by ZEA.

### Measurement Results of Fold Height, Myometrial Thickness, and Crypt Depth of Cecum Tissue

**FIGURE 2:**
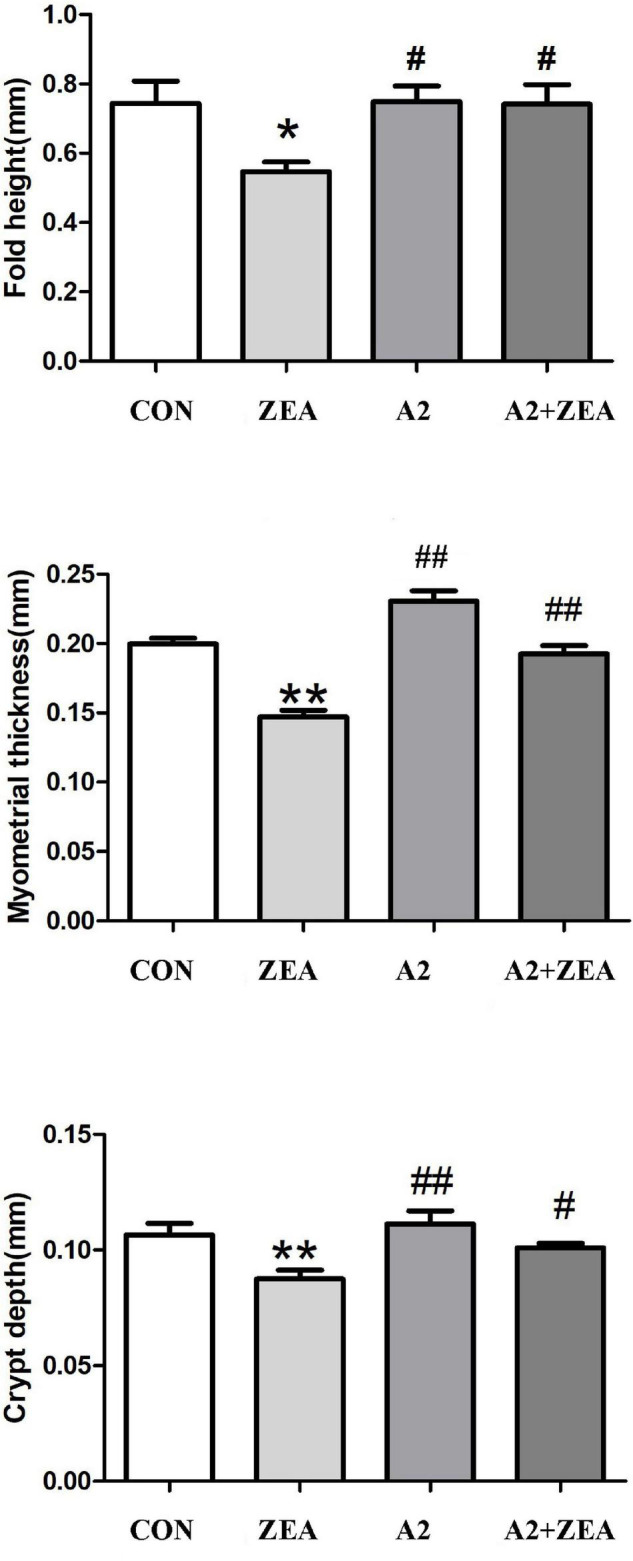
Measured fold height, myometrial thickness, and crypt depth of mice in each group. “*” denotes a significant difference compared with the CON group (*P* < 0.05), and “**” denotes an extremely significant difference compared with the CON group (*P* < 0.01). “#” represents a significant difference compared with the ZEA group (*P* < 0.05), “##” refers to an extremely significant difference compared with the ZEA group (*P* < 0.01).

Five mice were randomly selected from the CON group, ZEA group, A2 group, and A2 + ZEA group to measure the fold height, myometrial thickness, and crypt depth, respectively. In Figure 2, SPSS 20.0 and Graphpad Prism 5 software could be used for statistical analysis of the results. It was found that after feeding with ZEA, the fold height of cecum decreased remarkably compared with the CON group (*P* < 0.05), and the myometrial thickness and crypt depth decreased significantly (*P* < 0.01). However, when mice were treated with A2, compared with the ZEA group, the fold height of the A2 group showed a significant increase (*P* < 0.05); and the myometrial thickness and crypt depth were significantly increased (*P* < 0.01). Compared with the ZEA group, the fold height and crypt depth in the A2 + ZEA group were significantly declined (*P* < 0.05), and the myometrial thickness was significantly increased (P < 0.01). In conclusion, feeding A2 in mice could significantly alleviate the degree of ZEA damage to cecal tissue.

### Alcian Blue Staining Results of the Cecum

**FIGURE 3:**
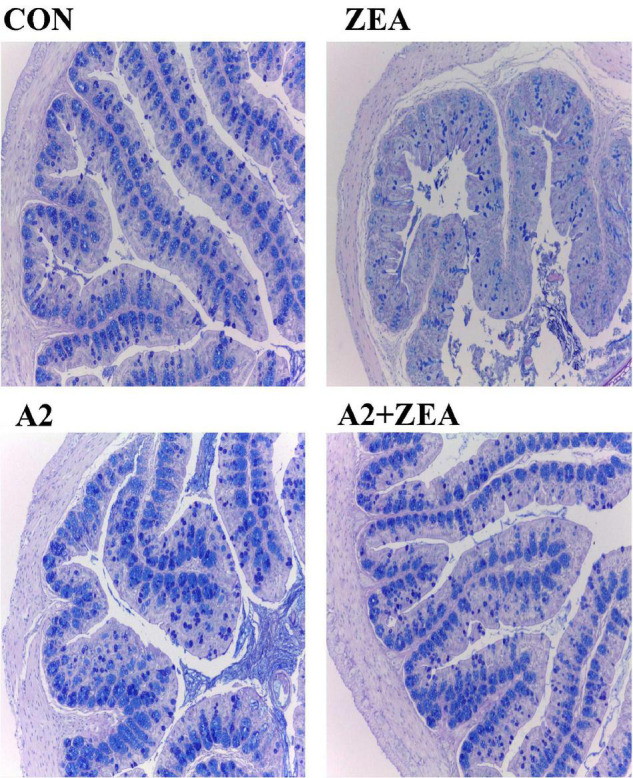
Results of Alcian blue staining.

Goblet cells are a kind of single-celled gland commonly distributed between the columnar epithelium of the mucosa of the small intestine. When goblet cells are mature, the cytoplasm was filled with mucinogen particles, which could secrete a large number of mucins, and the terminal sialic acid and sulfate lipids were acidic, thus forming a mucosal barrier to protect intestinal tissue (15). Therefore, in this experiment, the AB staining method was used to dye the acidic mucous cells in the cecum of mice blue to show the number of goblet cells, and then reflect the functional state of the cecum therein. As shown in Figure 3, the number of goblet cells in the ZEA group was significantly reduced, and the structure of cecum tissue was disorganized compared with the CON group. Compared with the ZEA group, the number of goblet cells in the A2 group and A2 + ZEA group increased significantly, which was not different from that in the CON group. These results indicate that A2 ameliorated ZEA-induced cecal injury and restored intestinal mucosal function in mice.

### Detection Results of Inflammatory Indicators in the Cecum of Mice

**FIGURE 4:**
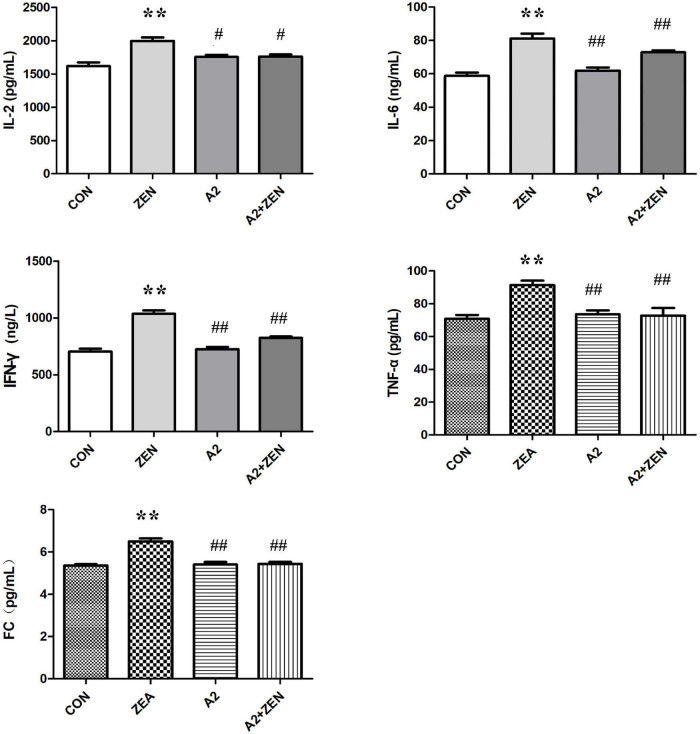
Test results of cecum verification indicators in each group included four groups: the data in the figure all come from three independent repeated tests. “**” means an extremely significant difference compared with the CON group (*P* < 0.01). “#” means a significant difference compared with ZEA (*P* < 0.05), “##” means an extremely significant difference compared with ZEA (*P* < 0.01).

As shown in Figure 4, compared with the CON group, expression levels of serum IL-2, IL-6, IFN-γ, TNF-α, and fecal FC in the ZEA group were significantly increased (*P* < 0.05); compared with the ZEA group, the levels of serum IL-2 in A2 and A2 + ZEA groups were significantly down-regulated (*P* < 0.05), and the levels of IL-6, IFN-γ, TNF-α, and FC were extremely significantly decreased (*P* < 0.01). In conclusion, the level of inflammation in the cecum of mice that fed A2 decreased significantly, and A2 could ameliorate the damage caused by ZEA to the cecum of mice.

### Detection Results of the Protein Expression of Inflammatory Factors by Western Blot Assay

**FIGURE 5:**
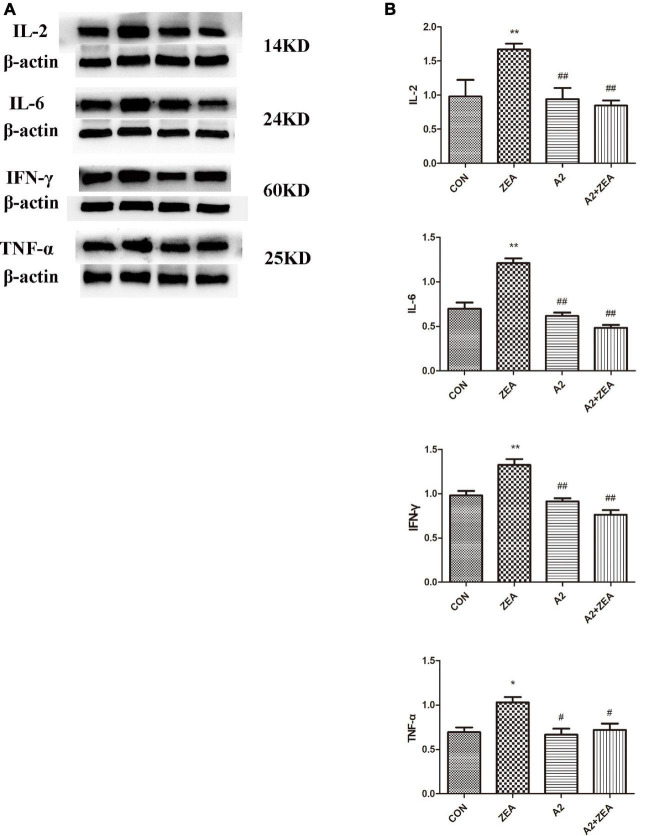
**(A)** Protein expression of cecal inflammation indicators in each group. **(B)** The data in the figure were all from at least three independently repeated trials. “*” means a significant difference compared with the CON group (*P* < 0.05), and “**” means an extremely significant difference compared with the CON group (*P* < 0.01). “#” means a significant difference compared with ZEA (*P* < 0.05), “##” means an extremely significant difference compared with ZEA (*P* < 0.01).

Figure 5A demonstrated the protein expression levels of inflammatory factors. The data were statistically analyzed, as shown in Figure 5B. Compared with the CON group, the expression levels of inflammatory cytokines IL-2, IL-6, and IFN-γ in the ZEA group were significantly up-regulated (*P* < 0.01), and the expression level of TNF-α was significantly increased (*P* < 0.05). On the contrary, compared with the ZEA group, the expression levels of inflammatory cytokines IL-2, IL-6, and IFN-γ in the A2 + ZEA group were significantly decreased (*P* < 0.01), and the expression level of TNF-α was significantly decreased (*P* < 0.05). These results indicated that the addition of A2 probiotics could effectively alleviate ZEA-induced inflammatory damage to the cecum.

### Detection Results of Short Chain Fatty Acids in Mouse Feces

**FIGURE 6:**
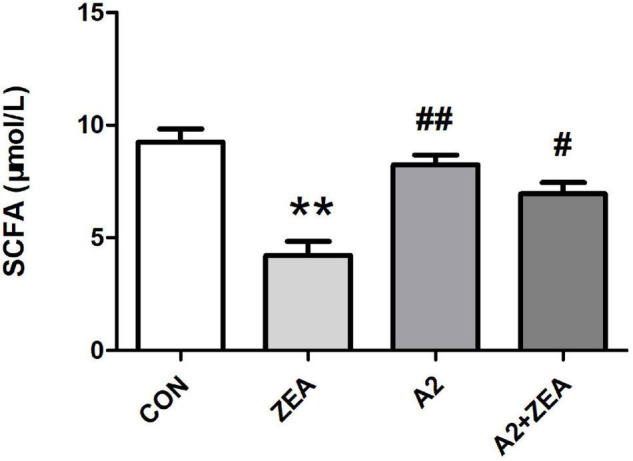
SCFA detection of mice feces in each group. “**” means an extremely significant difference compared with the CON group (*P* < 0.01). “#” means a significant difference compared with ZEA (*P* < 0.05), “##” means an extremely significant difference compared with ZEA (*P* < 0.01).

As shown in Figure 6, SCFA contents in feces of mice in the ZEA group decreased remarkably compared with that in the CON group (*P* < 0.01). Meanwhile, the SCFA content of mice was significantly changed after feeding A2, and the SCFA content of the A2 group was significantly increased compared with the ZEA group (*P* < 0.01). The SCFA content of the A2 + ZEA group was significantly increased (*P* < 0.05) compared with the ZEA group. In conclusion, mice fed ZEA could notably decrease the SCFA content of feces, so this change may be one of the causes of ZEA toxicity. In addition, the increase in SCFA content may be the key reason for A2 to improve ZEA-induced intestinal inflammation.

### Analysis of α and β Diversity

**FIGURE 7:**
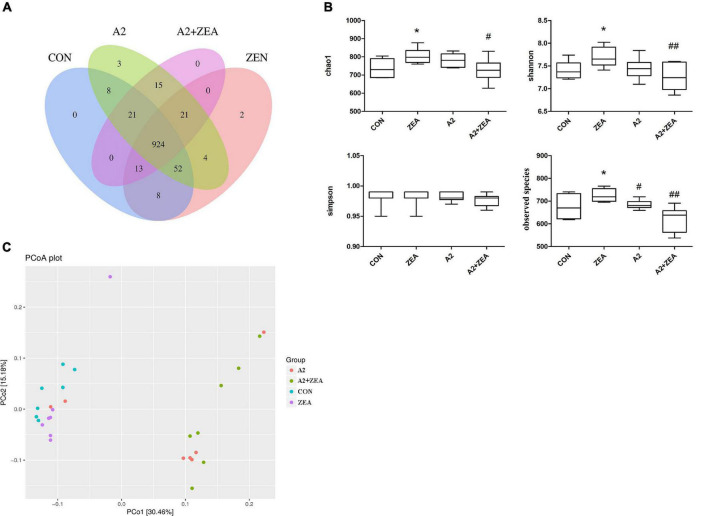
Analysis of the diversity of α and β by OTU distribution map **(A)**, Chao1, Shannon, Simpson, and observed species **(B)** represented α diversity. β diversity analysis was described by PCOA chart **(C)**. “*” means a significant difference compared with the CON group (*P* < 0.05). “#” means a significant difference compared with ZEA (*P* < 0.05), “##” means an extremely significant difference compared with ZEA (*P* < 0.01).

In the experiment, 16S rDNA sequencing analysis was conducted on the microorganisms in the cecum of mice. Sequences with sequence similarity of more than 97% in the four groups of samples were clustered into OTUs, and the sequence and representative sequence (OTU) of each cluster was obtained. Finally, OTU abundance values that were lower than 0.001% of the total samples were removed and its abundance was counted and assessed downstream. As illustrated in Figure 7A, the Venn diagram depicts the number of OTUs in each group, in which a total of 1071 OTUs was counted. There were 29 and 27 special OTUs in the CON group and ZEA group, respectively. For the ZEA group and A2 + ZEA group, the number of special OTUs was 66 and 36, respectively. In conclusion, ZEA changed the species of intestinal microflora in the cecum of mice, and the composition of microflora exhibited a significant change in the process of A2 alleviation of ZEA-induced damage to the cecum.

Further to determine the richness and uniformity of species in the samples, alpha diversity analysis was undertaken in this experiment, as shown in Figure 7B. Data analyses of Chao1, Shannon, Simpson, and observed species indices were conducted using GraphPad Prism 9. It is known that Chao1 and observed species can elucidate the number of OTU species in the sample, i.e., richness, and Shannon and Simpson indices reflect the evenness of species abundance in sample 20. The results showed that the richness and evenness of intestinal flora changed significantly after treatment with ZEA and A2. For example, Chao1, observed species, and Shannon in the ZEA group were significantly increased compared with the CON group (*P* < 0.05). Compared with the ZEA group, species richness and evenness of the A2 + ZEA group decreased significantly: Chao1 decreased (*P* < 0.05), and Shannon and observed species decreased significantly (*P* < 0.01). Therefore, it was inferred that the mechanism of action of A2 and ZEA may be closely related to the variation of richness and uniformity in the intestinal flora, which has important implications for further revealing the detoxification mechanism of ZEA.

The β diversity analysis in this experiment was carried out based on the unweighted Unifrac distance matrix in the four communities, and the differences between the sample groups were further evaluated by PCoA, as shown in Figure 7C. The results showed that there were significant differences in the microbial composition among the four groups, especially between the mice that ate A2 strain (A2 and A2 + ZEA groups) and the mice that did not eat A2 strain (CON and ZEA groups). The results indicated that A2 could significantly affect the microbial composition in the cecum of mice.

### Analysis of Differences Between Groups

**FIGURE 8:**
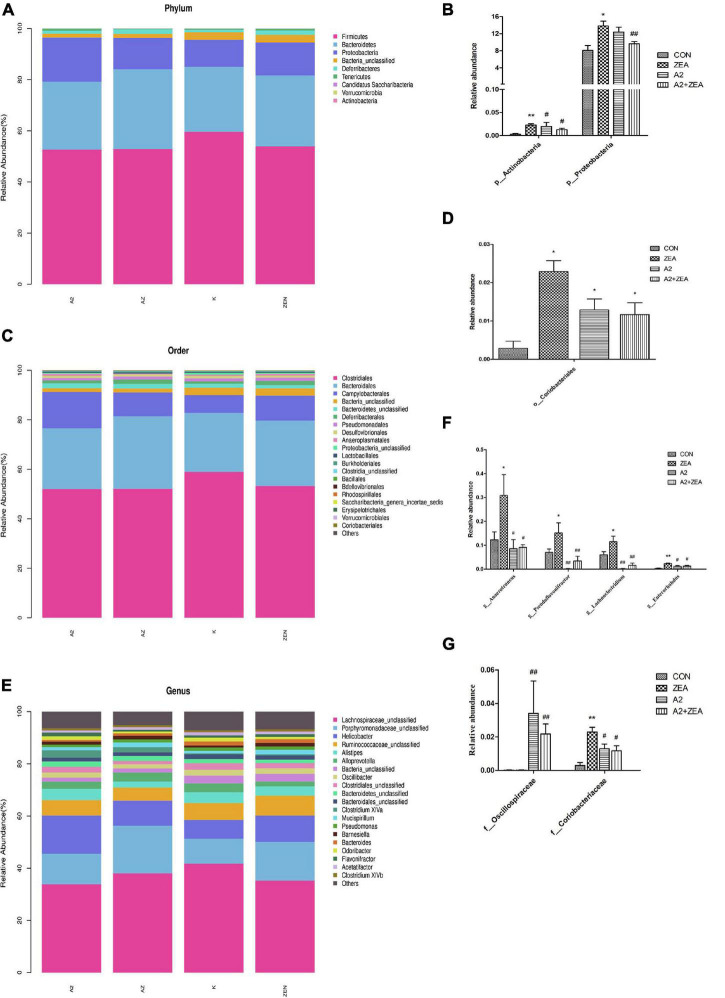
Annotation and classification of phyla **(A)**, order **(C)** and genus **(E)**. The ordinate represents the relative abundance of species at each classification level, and the abscissa denotes each classification level. Histograms of species showing significant differences at each rank include phyla **(B)**, order **(D)**, family **(G)**, and genus **(F)**. The abscissa marks the microbial community, and the ordinate marks the abundance of the community. “*” means a significant difference compared with the CON group (*P* < 0.05), and “**” means an extremely significant difference compared with the CON group (*P* < 0.01). “#” means a significant difference compared with ZEA (*P* < 0.05), “##” means an extremely significant difference compared with ZEA (*P* < 0.01).

In this experiment, in order to annotate and make abundance comparison of the species in the four groups of samples, the relative abundance of four groups of samples was calculated, and the differences and similarities among the top 20 species were examined. Abundance bars at phylum, order, and genus levels are illustrated in Figure 8, and some species showing significant differences at abundance levels are listed.

At the phyla level (Figure 8A), the most representative phyla in the CON group include *Firmicutes* (54.73%), *Bacteroidetes* (27.74%), *Proteobacteria* (13.22%), *Deferribacters* (1.42%), and *Tenerictes* (0.56%), and these six phyla account for more than 97% of the total abundance. In Figure 8B, the relative abundance of *Actinobacteria* in the CON group was found to account for only 0.01% overall, but it still showed an obvious trend in different treatment groups. For example, the ZEA group increased significantly compared with the CON group (*P* < 0.01), and the A2 and A2 + ZEA groups decreased significantly compared with the ZEA group (*P* < 0.05). Therefore, *Actinobacteria* may play a critical role in the process of intestinal inflammation induced by ZEA. Combined with the previous test results, A2 could significantly reduce the inflammation triggered by *Actinobacteria*. The relative abundance of *Proteobacteria* in the ZEA group was significantly higher than that in the CON group (*P* < 0.05); Compared with the ZEA group, the relative abundance of *Proteobacteria* in A2 group showed a decreasing trend, but the effect was insignificant; Compared with the ZEA group, the A2 + ZEA group decreased significantly (*P* < 0.01). A2 and ZEA were found to reduce the abundance of pathogenic *proteobacteria* synergistically. In conclusion, at the phyla level, A2 could effectively improve ZEA-induced intestinal inflammation, which may be associated with the reduction of the relative abundance of *Actinobacteria* and *Proteobacteria* in the cecum.

At order level (Figure 8C), the most representative orders in CON include *Clostridiales* (58.9%), *Bacteroidales* (23.86%), and *Campylobacterales* (7.18%), of which the relative abundance of these four phyla exceeded 90%, and the relative abundance of other species was less than 2%. In Figure 8D, it was found that the relative abundance of *Coriobacterials* in the CON group was low, specifically 0.01%, but this strain showed significant differences in the four groups. *O_Coriobacteriales* was *p_ Actinobacteria* (a subordinate branch of *Actinobacteria*), and the changes in abundance of the two bacteria were similar. Compared with the CON group, *Coriobacteriales* in the ZEA group increased significantly (*P* < 0.05). This finding could prove that ZEA-induced intestinal inflammation was closely related to the significant increase of the relative abundance of *Coriobacteriales*. The relative abundance of *Coriobacteriales* in the A2 group and the A2 + ZEA group decreased significantly (*P* < 0.05) compared with the ZEA group. Combined with the previous test results, *Coriobacteriales* may play an important role in cecal inflammation as a pathogen. The combined treatment of A2 and ZEA could reduce the abundance of *Coriobacteriales* and ameliorate the inflammatory damage caused by ZEA.

Figure 8G illustrates the species in the family that indicates significant differences in relative abundance, including *f_Oscillospiraceae* and *f_Coriobacteriaceae*. *f_Coriobacteriae* is the subordinate classification of o_*Coriobacterials*, and the two showed similar trends. The relative abundance of *Oscillospiraceae* was less in the CON and ZEA groups, but greater in the A2 group. Specifically, the relative abundance of *Oscillospiraceae* in the A2 group and A2 + ZEA group markedly increased compared with the ZEA group (*P* < 0.01). The decrease of inflammation in the cecum may be attributed to the increase in *Oscillospiraceae* abundance therein caused by A2.

In the genus level (Figure 8E), the most representative genera in CON include *Helicobacter* (7.18%), *Alistipes* (4.19%), *Alloprevotella* (3.37%), and the relative abundance of other species was less than 3%. As illustrated in Figure 8F, the genera showing significant differences in relative abundance include *Anaerotruncus*, *Pseudoflavonifractor*, *Lachnoclostridium*, and *Enterorhabdus*, and the trends therein are similar, for example, compared with the CON group, the relative abundance of *Anaerotruncus*, *Pseudoflavonifractor*, and *Lachnoclostridium* in the ZEA group increased significantly (*P* < 0.05), and *Enterorhabdus* increased significantly (*P* < 0.01); Compared with the ZEA group, the relative abundances of *Anaerotruncus* and *Enterorhabdus* decreased significantly (*P* < 0.05), and *Pseudoflavonifractor* and *Lachnoclostridium* showed a significant decrease in relative abundance (*P* < 0.01). The increase of the relative abundance of *Anaerotruncus*, *Pseudoflavonifractor*, *Lachnoclostridium*, and *Enterorhabdus* may play a pathogenic role in the process of cecal inflammation induced by ZEA, and A2 treatment could decrease the abundance of these pathogens and alleviate the inflammatory injury caused by ZEA to the cecum.

## Discussion

Previous research found that the intestinal mucus secreted by goblet cells in the intestinal epithelium was significantly disturbed by inflammatory diseases such as celiac disease and inflammatory bowel diseases, and significant changes occurred in the production and composition of intestinal mucus (16). When broiler and mouse models were exposed to DON, ZEA, FB1, and AFB1, an increase/proliferation of goblet cells could be observed and an increase in mucin secretion was induced (17). Normally, excessive mucin secretion can lead to depletion of goblet cells and destruction of the mucus layer (18). At this time, the normal function of the intestinal tract is severely disrupted and the balance is broken. This may result in the destruction of tight junction proteins, the increase of intestinal permeability, the increase of inflammatory factors, cell necrosis, the inhibition of cell proliferation, and the up-regulation of immunoglobulin, eventually triggering inflammation and intestinal damage (19).

The results agree with the results of the present study, the number of goblet cells in the cecal structures of mice was significantly decreased in the ZEA group, while the number of goblet cells returned to normal levels in the A2 + ZEA group. This was ascribed to hyperplasia and severe depletion of goblet cells in the ZEA group, which eventually caused the destruction of the mucus layer and intestinal inflammation, while the extents of intestinal damage and inflammation were notably reduced after feeding with the A2 strain.

When the mycotoxin enters the GIT, some will be absorbed quickly, some will go further into the intestinal tract and participate in the systemic circulation process, then participate in the interaction of the intestinal microbiome, thus intestinal microbiome was found to exert important effect on mediating intestinal toxicity (20). For example, ochratoxin A (OTA) permanently eliminates *Lactobacillus reuteri* in the human colon, a fungus that is known to have a positive effect on intestinal inflammation, infection, and immune response (21). According to reports, *Firmicutes*, *Bacteroidetes*, *Proteobacteria*, and *Actinobacteria* (at phylum level) are the predominant bacterial groups. Chronic intestinal inflammation is likely to occur when the proportion of bacteria groups is unbalanced (22). For example, in patients with end-of-stage renal disease (ESRD), the relative abundance of *Proteobacteria* significantly increased, while the abundance of *Firmicutes* and *Actinobacteria* remarkably decreased (23). A significant increase in the relative abundance of pathogenic bacteria *Actinobacteria* was found in the GIT of patients with early chronic kidney disease (CKD) and patients with cancer (24, 25). It has also been reported that the high abundance of *Proteobacteria* in mice will also lead to local or systemic inflammation and metabolic dysfunction, which greatly increases the chance of chronic colonic inflammation (26). Similar results were also found in this experiment. For example, compared with the ZEA group, the abundance of pathogenic bacteria *Actinobacteria* and *Proteobacteria* in the A2 + ZEA group markedly decreased. These fungi were involved in the inflammatory injury induced by ZEA, and the decrease of their relative abundance was closely related to the alleviation of intestinal inflammation caused by ZEA by A2.

Another reason for the intestinal inflammatory reaction to occur is due to the changes in intestinal SCFA, for example, OTA after disrupting the balance in intestinal flora demonstrated significantly reduced concentrations of acetic acid, butyric acid, and SCFA and severe intestinal damage in a human colon model (27). It can be seen that mycotoxins caused severe harm to intestinal health and the production of fermentation products and imparted a serious effect on the animal body. Similar results were also found in this study. The SCFA content in the ZEA group was significantly decreased compared with the CON group, while the SCFA content was significantly reduced after ZEA-treated mice were fed A2, and the intestinal inflammation of mice was significantly decreased. This effect arises because SCFA plays an important role in intestinal epithelial barrier function in conjunction with tight junction proteins (28). It has been reported that butyrate can elevate the expression of Claudin-1 and ZO-1 in the colon, promoting the redistribution of Occludin to enhance the barrier function of the intestinal tract, and inhibiting intestinal inflammation (29. This is because butyrate can stimulate the activation of intracellular AMP-activated protein kinase (AMPK) and effectively blocks the effect of permeability (30).

It has been proved that SCFA plays a role in inflammation and immune response. It is now known that the synthesis of SCFA is related to G-protein coupled receptors (GPRCs) and histone deacetylases (HDACs). SCFA can suppress lipopolysaccharide (LPS)-induced inflammation through these two mechanisms (31). For example, butyrate and propionate decreased the expression levels of TNF and nitric oxide synthase (NOS) in monocytes induced by LPS; acetate inhibited LPS-induced TNFα secretion from human and mouse monocytes *via* the FFAR receptor; butyrate inhibits NF-κB and histone deacetylation (HDAc) in macrophages, both of which are involved in inflammatory and immune signaling (27, 32). In conclusion, SCFA affected by intestinal microorganisms has an important effect on the regulation of cecal inflammation.

Another aspect of the influence of SCFA on intestinal inflammation is that it is able to affect the production of mucus in the gut. Intestinal mucus is not only an energy source for gut microbes but also acts as a chemical barrier, protecting the body from toxins, pathogens, and antibodies (33). Mucin secretion was reported to increase in a dose-dependent manner and the number of Goblet cells was observed to increase *via* increasing the levels of acetate and butyrate in a mouse colon model (35). It was found that the regulation of goblet cells by butyrate is mediated by galectin-1 gene stimulation of the regulatory glycosylation enzyme that is an enzyme found to have protective effects in experimental research into colitis (35). In addition, this sugar can also be used as a microbial growth substrate and adhesion site to participate in the interaction between the intestinal epithelium and microorganisms, which further confirms that SCFA can participate in the regulation of intestinal microorganisms by regulating goblet cells in the intestinal epithelium. For example, butyrate enhanced the adhesion of the beneficial bacteria *Lactobacillus* and *Bifidobacteria* in LS174T colonic epithelial cells and inhibited the adhesion and translocation of pathogenic bacteria *Escherichia coli* (36). SCFA can affect the secretion of intestinal mucus at the molecular level, regulate the composition of the microbial community, and thus reduce intestinal inflammation.

Maintaining the homeostasis of intestinal microbiota plays a key role in maintaining human and animal health and preventing diseases. For example, inflammatory bowel diseases, irritable bowel syndrome (IBS), Type 2 diabetes (T2D), obesity, autoimmune disorders, and cancer have been identified as being caused by the imbalance of intestinal microbiota and the reduction in the number of bacteria producing SCFA metabolites (37). It is known that the content of SCFA is not only affected by the species, composition, environment, diet, and genetic factors pertaining to intestinal microorganisms, but also may be affected by probiotic additives (38).

Although the present research focuses on the involvement of A2 in the regulatory effect of SCFA on ZEA-induced cecal inflammatory injury in mice, its mechanism of action warrants further investigation. Use of probiotics to regulate intestinal flora, regulate the content of SCFA in the intestinal tract, and prevent the inflammatory injury of mycotoxins should prove fruitful avenues for further research.

## Conclusion

The experiment proved that *Bacillus velezensis* A2 could improve the intestinal inflammatory damage caused by Zearalenone to the cecal of mice. Through the analysis of 16S rDNA sequencing technology to detect the changes of intestinal flora in mouse cecal and the content of SCFA in mouse feces, it is proved that *Bacillus velezensis* A2 can alleviate the inflammatory damage caused by Zearalenone by regulating intestinal flora and SCFA.

## Data Availability Statement

The datasets presented in this study can be found in online repositories. The names of the repository/repositories and accession number(s) can be found in the article/supplementary material.

## Ethics Statement

The animal study was reviewed and approved by the experimental Animal Care Ethics Committee of Shenyang Agricultural University and its full name has been listed in 2.1 of the manuscript. Here is the number of animal experiment approval (Permit No. 264SYXK2011-0001, September 2018).

## Author Contributions

JCa, NW and JCh: investigation. JCa: writing – original draft preparation. JCh, EN, AW, MV and WW: writing – review and editing. ML, WW, and KK: supervision. All authors have read and agreed to the published version of the manuscript.

## Conflict of Interest

The authors declare that the research was conducted in the absence of any commercial or financial relationships that could be construed as a potential conflict of interest. The reviewer XW declared a shared affiliation with one of the authors AW to the handling editor at the time of review.

## Publisher’s Note

All claims expressed in this article are solely those of the authors and do not necessarily represent those of their affiliated organizations, or those of the publisher, the editors and the reviewers. Any product that may be evaluated in this article, or claim that may be made by its manufacturer, is not guaranteed or endorsed by the publisher.

## Footnotes

1
https://www.ncbi.nlm.nih.gov/sra/PRJNA773604
